# CHARACTERISTICS OF THE GAIT INITIATION PHASE IN OLDER ADULTS WITH DIABETIC PERIPHERAL NEUROPATHY

**DOI:** 10.1093/geroni/igz038.1765

**Published:** 2019-11-08

**Authors:** Gu Eon Kang, Hung Nguyen, Mohsen Zahiri, He Zhou, Changhong Wang, Rahul Goel, Bijan Najafi

**Affiliations:** Baylor College of Medicine, Houston, Texas, United States

## Abstract

Impairment in steady-state gait in older adults with diabetic peripheral neuropathy (OADPN) is well-known, however little attention has been paid to the gait initiation phase in which postural transitions occur from upright standing to steady-state gait. Given the risk of falls in the gait initiation phase in older adults, knowing its characteristics may be as important as steady-state gait. The aim of this study was to investigate kinematic characteristics of the gait initiation phase in OADPN compared to healthy older adults (HOA). Thirteen OADPN (72.9±6.1 years; 33.0±4.8 kg/m2), and 11 HOA (71.8±2.7 years; 26.5±4.3 kg/m2; no cardiovascular, neurological or orthopedic condition, no history of falling) performed gait on level ground for minimum 10 meters at self-selected comfortable speed. We collected kinematic data using five wearable sensors (LEGSysTM, BioSensics LLC, Watertown, MA) attached on the shanks, thighs and lower back. We used previously validated algorithm to analyze kinematic parameters for the gait initiation phase. Our statistical model showed that the number of steps, stride velocity, gait cycle time, double limb support and mediolateral center-of-mass sway during the gait initiation phase is significantly different between HOA (2.4±0.7 steps; 1.16±0.15 m/s; 1.12±0.10 seconds; 20.3±4.8%; 4.0±1.5°, respectively) and OADPN (4.0±2.1 steps; 0.92±0.29 m/s; 1.23±0.12 seconds; 29.2±10.3%; 7.0±2.9°, respectively) (all p<0.05). The results suggest that OADPN take more, slower and more unstable steps to reach steady-state gait from upright standing compared to HOA. The results also provide implications for needs to develop new interventions targeting the gait initiation phase in OADPN.

